# Case of a CD3 Negative Hepatosplenic T-Cell Lymphoma: Diagnostic and Therapeutic Challenges

**DOI:** 10.1155/2019/5315086

**Published:** 2019-06-27

**Authors:** Laura Alder, Scott Graupner, Guanhua Lai, Victor Yazbeck

**Affiliations:** ^1^Department of Medicine, VCU Health, 1201 E Marshall St, Richmond, VA 23298, USA; ^2^Department of Hematology Oncology, Massey Cancer Center, VCU Health, 401 College St, Richmond, VA 23298, USA; ^3^Department of Pathology, VCU Health, 1201 E Marshall St, Richmond, VA 23298, USA

## Abstract

Hepatosplenic T-cell lymphomas (TCLs) are a rare, aggressive subset of TCLs, accounting for less than 5% of all peripheral T-cell and natural killer (NK) cell lymphomas. We report the case of a CD3 negative hepatosplenic T-cell lymphoma in a 42-year-old female, who presented with left-sided abdominal pain. She underwent a liver biopsy that showed marked abnormal sinusoidal lymphoid infiltration. PET scan revealed increased splenic and pharyngeal lymph node uptake. Immunophenotype was remarkable for negative CD3, gamma delta T-cell receptor, and alpha beta-T-cell receptor expression. She received 6 cycles of DA-EPOCH, had primary refractory disease and then underwent palliative splenectomy secondary to painful necrosis. Then, she was started on pralatrexate as a single agent and then in combination with romidepsin as a potential bridge to an allogeneic stem cell transplantation from her sibling.

## 1. Background

Hepatosplenic T-cell lymphomas (HSTCLs) are a rare subtype, accounting for less than 5% of all peripheral T-cell and NK cell lymphomas. They have a very poor prognosis, with median survival less than 5 years and rare complete remissions [1]. They are characterized by cytotoxic T-cells infiltrating hepatosplenic sinus leading to hepatosplenomegaly as the most common clinical presentation. While the bone marrow is involved in two-thirds of cases, minimal nodal involvement is typical. Cytopenias, especially thrombocytopenia, are common with a predilection for developing hemophagocytic syndrome [2]. HSTCLs are predominantly seen in males with a median age of onset of 35 years [3]. Diagnosis is made by histologic examination and immunohistochemistry of a bone marrow biopsy and/or liver biopsy or splenectomy vs spleen biopsy. Tumors involve cytotoxic T-cells frequently expressing the gamma/delta T-cell receptor and the immunophenotype CD2+, CD3+, CD56+, and TIA1+ by flow cytometry analysis. Genetic features commonly detected by FISH analysis include isochromosome 7q and trisomy 8, while activating mutations of the JAK/STAT pathway have been reported [4]. Some of the main differential diagnoses to consider when making the diagnosis of HSTCL are aggressive natural killer cell leukemia, gamma/delta-expressing T-cell large granular lymphocytic leukemia (T-LGLL), reactive gamma/delta T-cell proliferations, and more rarely other T-cell lymphomas that may have gamma/delta expression. HSTCL have a dismal prognosis with a mean survival of less than a year and poor response to conventional chemotherapy. New targeted therapies such as pralatrexate, duvelisib, and romidepsin are emerging as potential treatment options as a bridge for allogenic HSCT, which remains the only potential curative treatment modality [5].

## 2. Case Presentation

**Figure 1:**
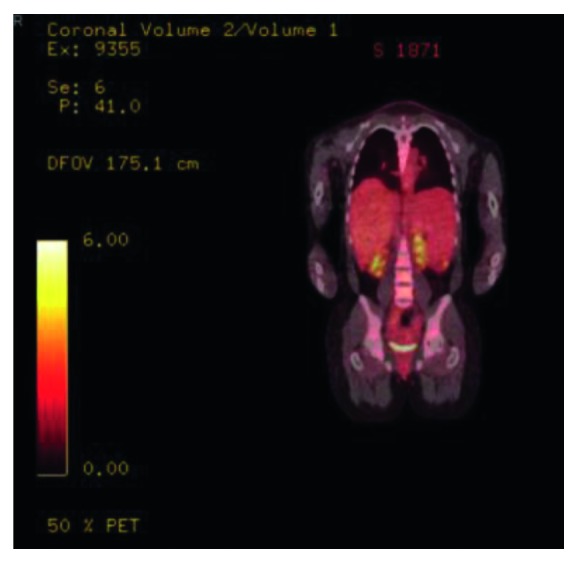
PET scan, initial coronal section at presentation, showing marked splenomegaly. The spleen measured approximately 20 cm in length with relatively focal areas of increased radiotracer uptake in the splenic parenchyma with max SUV 4.4, compared to background max SUV throughout the spleen ranging 2.5–3.0. Background radiotracer uptake throughout the liver was very slightly greater than of the spleen, 2.7–3.2. There was also a focus of asymmetric increased radiotracer uptake in the left parapharyngeal soft tissues with associated soft tissue thickening on CT images and maximum SUV 3.5.

A 42-year-old African‐American female with a past medical history significant for ductal carcinoma in situ diagnosed five years ago, treated with radiation and lumpectomy, presented with a two months history of progressively worsening left-sided abdominal pain and distension. Lab work showed anemia (hemoglobin of 7.3) and thrombocytopenia (platelets of 126) with leukocytosis (WB of 16.5). Due to concern for hemophagocytic lymphohistiocytosis (HLH), additional labs were drawn; her fibrinogen was 339 mg/dL, triglycerides 217 mg/dL, and ferritin 550 ng/mL, which overall did not support the diagnosis. Imaging studies showed marked splenomegaly and mild hepatomegaly. PET scan showed increased splenic and pharyngeal lymph node uptake (Figure 1).

**Figure 2:**
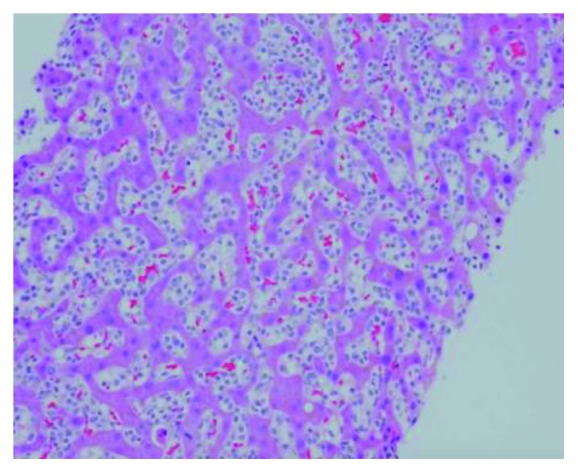
H&E staining of the liver core biopsy shows abnormal diffuse sinusoidal lymphoid infiltration. The lymphocytes are small to intermediate in size with clear cytoplasm and devoid of azurophilic granules, 20x.

**Figure 3:**
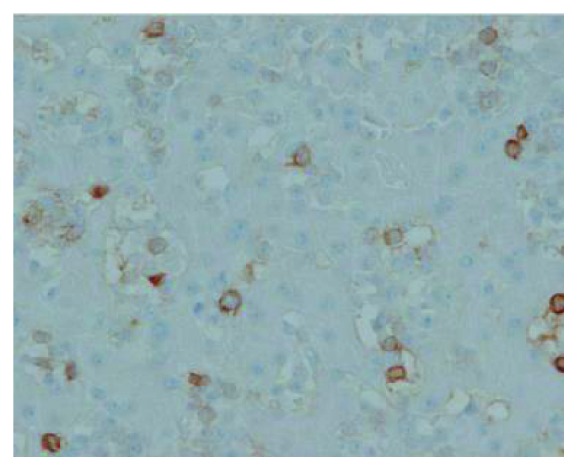
Immunohistochemistery for CD3 shows the abnormal lymphocytes are negative for CD3. Rare scattered T-cells are highlighted in the background, 40x.

Her liver biopsy showed infiltration by abnormal lymphocytes (Figures 2 and 3) as did her bone marrow biopsy. The flow cytometry study performed on bone marrow aspirate demonstrated 53% abnormal lymphocytes with a phenotype of CD2+ CD3− CD7+ CD5− CD4− CD8− CD56+ CD57− CD16− TCRa/b− TCRg/d−, which was interpreted as NK cell population. Immunohistochemistery for CD3 performed on live core biopsy confirmed the abnormal lymphocytes are CD3 negative. Notwithstanding these abnormal immunophenotypes, further workup including Epstein–Barr virus (EBV) and T-cell receptor (TCR) gene rearrangement studies demonstrated negative EBV infection and positive monoclonal TCR gene rearrangement, which support the diagnosis of stage IV HSTCL with liver and bone marrow involvement. In order to differentiate between NK and T-cell lymphoma, TCR rearrangement studies are helpful like in this case as they can help finalize the diagnosis as HSTCL. The lack of evidence of any EBV infection in her biopsies or her serum DNA was not supportive of involvement by NK-cell leukemia.

Given her symptoms, she was started on prednisone for 1-2 weeks prior to completing 6 cycles of DA-EPOCH administered at dose level +1 that could not be escalated due to cytopenia. After 4 cycles, her interim PET scan showed diffuse hypermetabolic activity of the axial and appendicular skeleton, attributed to super expansion of the bone marrow secondary to chemotherapy. There was no suspicious lymphadenopathy of the chest, abdomen, and pelvis. She tolerated the cycles fairly well, complicated by one inpatient hospitalization for neutropenic fever with negative workup. The goal was to complete the 6 cycles of DA-EPOCH with positive response to allow for allogeneic HSCT if chemosensitive, ideally in CR1.

**Figure 4:**
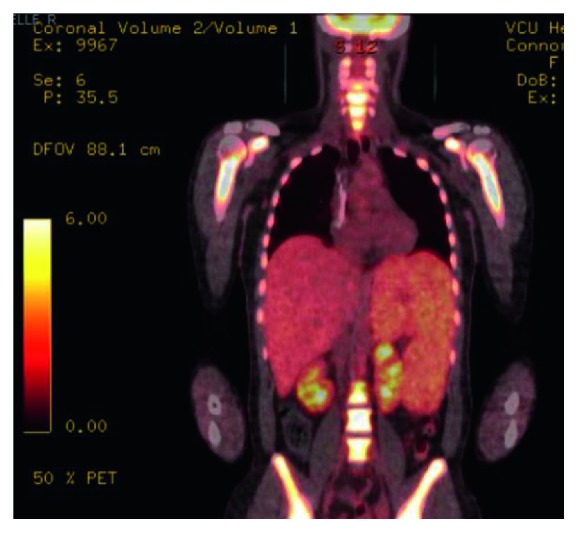
Pet scan 4 weeks after 6 cycles of DA-EPOCH showed massive splenomegaly. The spleen measures up to 27.7 cm in length with pronounced hypermetabolic activity throughout the spleen with a maximal SUV of up to 9.8. There are new peripheral wedge-shaped areas of photopenia consistent with infarcts. There is a pronounced mass effect on the stomach and left kidney from the massive splenomegaly. There is ongoing hepatomegaly as well, with the right hepatic lobe measuring 23.1 cm in length compared to 22.0 previously. Hepatic radiotracer uptake is homogenous but decreased from what would normally be expected (the average maximum SUV is approximately 1.9). This diffusely reduced radiotracer uptake could be secondary to a sequestration of radiotracer in the spleen. The previously described area of low-grade uptake in the left parapharyngeal soft tissues now demonstrates a maximal SUV of 2.0, previously 3.5.

**Figure 5:**
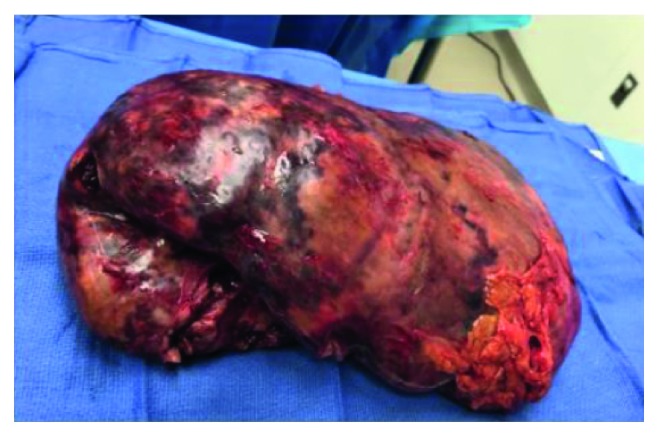
Patient underwent open splenectomy. Pathology reported a hepatosplenic T-cell lymphoma diffusedly involving a markedly enlarged spleen, 4025 grams, 29.5 × 22.0 × 10.9 cm. The capsule is tan, remarkable for adhesions and multiple defects ranging in size from 3.7 × 1.0 cm to 16.5 × 11.5 cm. The specimen is remarkable for three possible hilar nodes ranging in size from 1.2 × 0.7 × 0.5 cm to 4.5 × 2.1 × 2.0 cm. Sectioning through the spleen reveals gray-white to yellow hemorrhagic lobular cut surfaces. No definitive grossly unremarkable splenic parenchyma can be identified.

Unfortunately, her PET scan 4 weeks after her 6th cycle of DA-EPOCH showed she was primary refractory. Imaging also showed a new splenic infarct with worsening splenomegaly (Figure 4). Her labs at that time were significant for a WBC of 19.8, hemoglobin of 7.5, and platelet count of 77. Her liver function enzymes were within normal limits, except for an LD of 1452. Her main complaint was severe abdominal pain, with physical exam findings of tender splenomegaly occupying the whole left hemiabdomen, extending up to her pelvis. Given her symptoms and cytopenias thought to be worsened by her splenomegaly, she underwent an open splenectomy (∼9 lbs), as shown in Figure 5. Her recovery was complicated by small bowel injury and a left-sided pleural effusion.

The original plan was for salvage therapy with the combination of pralatrexate and romidepsin as a bridge to an allogeneic HSCT from her 10/10 HLA-match sister, but given her counts and performance status, we started her on single agent pralatrexate 10–20–30 mg/m^2^, of which she received 4 doses. Repeat PET scan showed disease progression, but given improvement in her performance status and counts, she was started on pralatrexate 25 mg/m^2^ and romidepsin 12 mg/m^2^·q for 2 weeks (days 1 and 15 of a 28-day treatment cycle).

Unfortunately, a few months after initiating combination therapy, she was hospitalized with a nosebleed, pancytopenia, and neutropenic fever. This progressed into worsening multiorgan failure of which she expired.

## 3. Discussion

This case highlights the diagnostic challenges in differentiating CD3 negative HSTCL from NK-cell neoplasm, which is crucial for guiding therapy. While her liver biopsy was suggestive of an NK-cell neoplasm given the presence of NK-cells and CD3 negativity, her clinical presentation was highly suggestive of HSTCL. The characteristic immunophenotype for HSTCL are CD2^+^/CD3^+^, CD5^−^, and CD4^−^/CD8^−^. Aggressive NK-cell leukemias share a similar immunophenotypic profile with HSTCL, with a negative surface CD3. HSTL is characterized by clonal rearrangement of TCR genes, whereas in aggressive NK-cell leukemia, the genes are typically in germline configuration [6], making TCR arrangement studies an important tool for reaching the correct diagnosis. Furthermore, aggressive NK-cell leukemia is EBV driven, whereas there is no association with EBV and HSTCL. Our patient was negative for EBV infection. It is essential to differentiate between the two entities since the treatment vary significantly. NK-cell lymphomas in the limited stage are usually treated with radiation either preceding or in combination with chemotherapy, which has been shown to improve overall survival. NK-cell lymphomas are generally resistant to CHOP-based chemotherapy regimens but are in many cases sensitive to asparaginase-containing regimens such as SMILE (dexamethasone, methotrexate, ifosfamide, L-asparaginase, and etoposide). Advanced stage NK-cell lymphomas are generally treated with aggressive asparaginase-containing regimens such as SMILE alone [7]. HSTCL cancers on the other hand are usually treated with anthracycline-based aggressive chemotherapy regimens such as DA-EPOCH followed by stem cell transplantation at first remission.

HSTCL is a rare subtype of PTCL, carries a poor prognosis, and has no single standard of care regimen. One of the major challenges for studying this disease is the rarity of the disease itself, which poses challenges in collecting enough data in order to assess efficacy of current therapies and conduct prospective clinical trials. Another challenge is the difficulty in developing risk stratification treatment algorithms that guide therapy. This patient was started on DA-EPOCH with the intention to consolidate with an allogeneic stem cell transplantation given that her sister is a 10/10 match. Allogenic HCT is considered the ultimate curative therapy. In a recent study, 54 patients with HSTCL that underwent allogeneic stem cell transplantation had an improvement in the median overall survival (68 months) compared to historical control (6–11 months) [8, 9]. Given her primary refractory disease, she underwent splenectomy, in order to improve her symptoms and cytopenias, with the hope to increase her count in order to tolerate her salvage therapy with single agent pralatrexate and then in combination with romidepsin [10]. Pralatrexate and romidepsin have shown a broad spectrum of activity across several T-cell histological subtypes, suggesting unique mechanism of actions, and have been increasingly used in all subtypes of peripheral T-cell lymphomas, including HSTCL, at relapse [11]. Case reports already suggest high efficacy with these novel agents in HSTCL. A recent phase 1 study showed that the combination of romidepsin and pralatrexate was well tolerated and resulted in an impressive overall response rate of 71% (10/14 patients) in patients with peripheral T-cell lymphomas including HSTCL [12].
